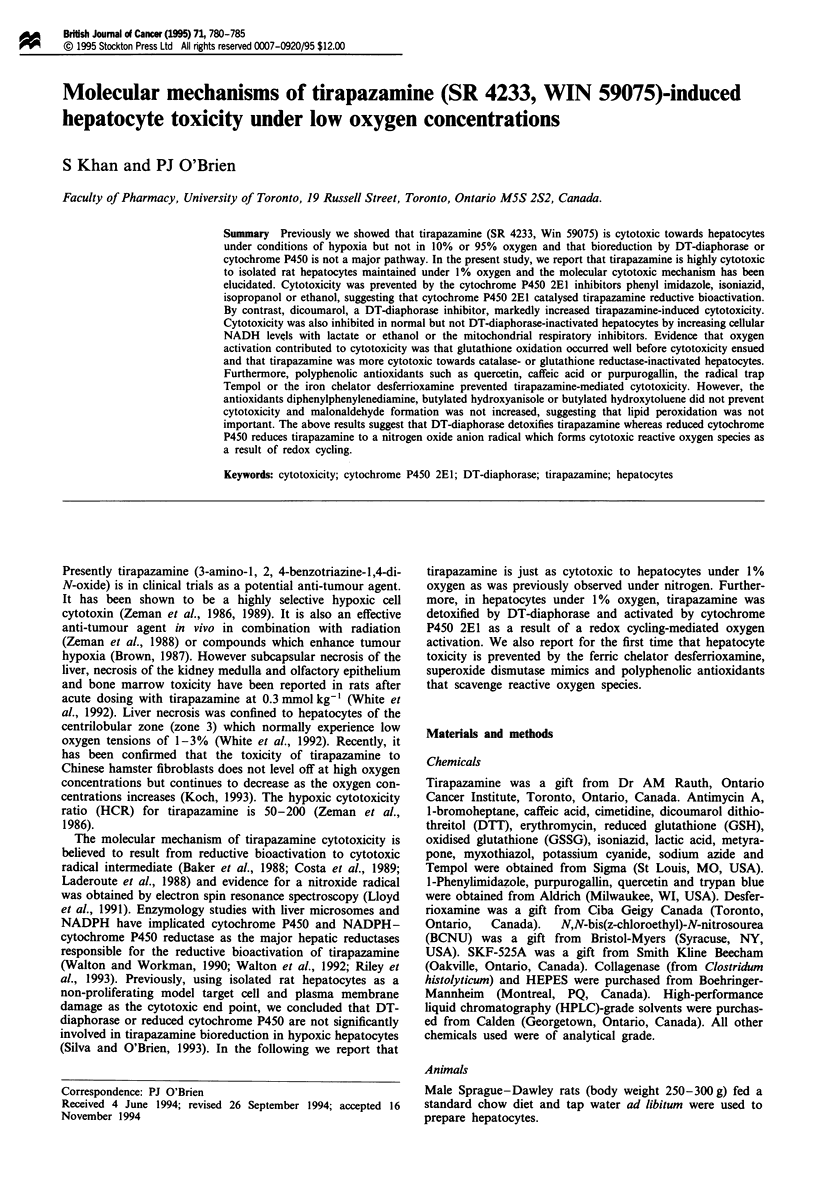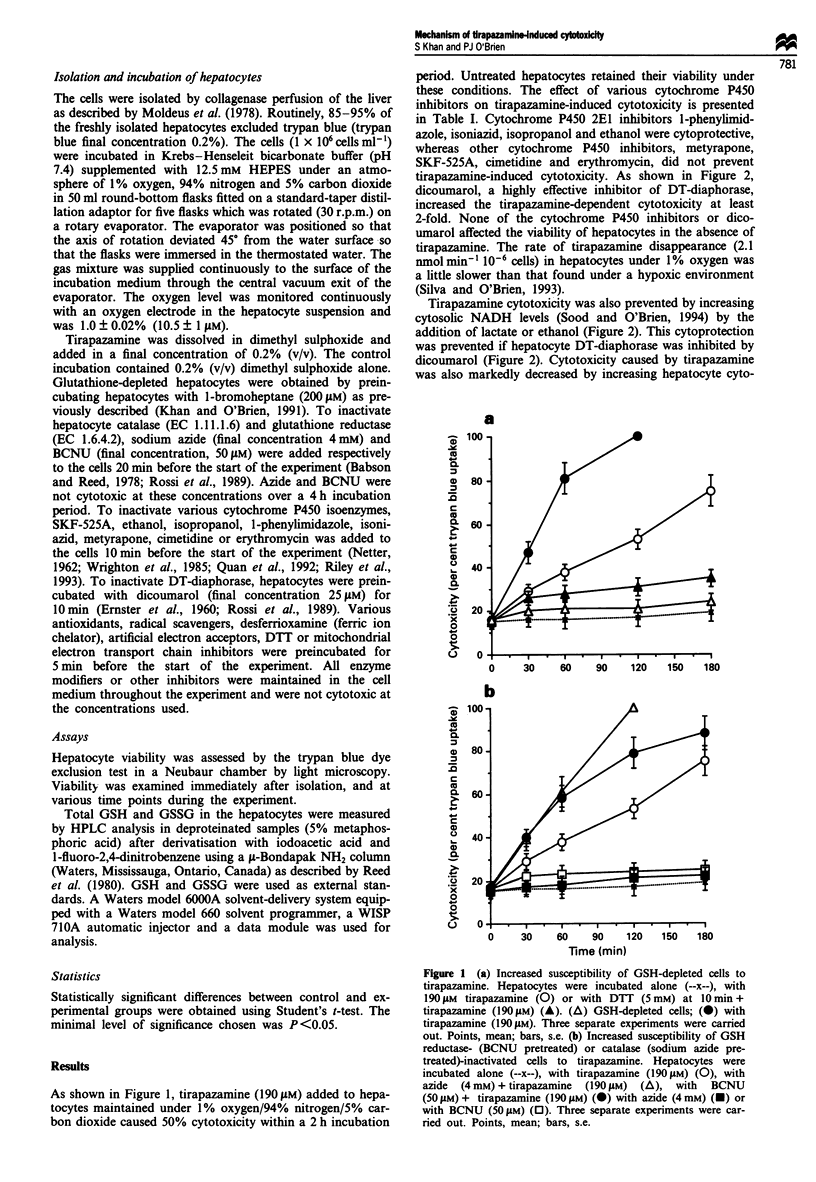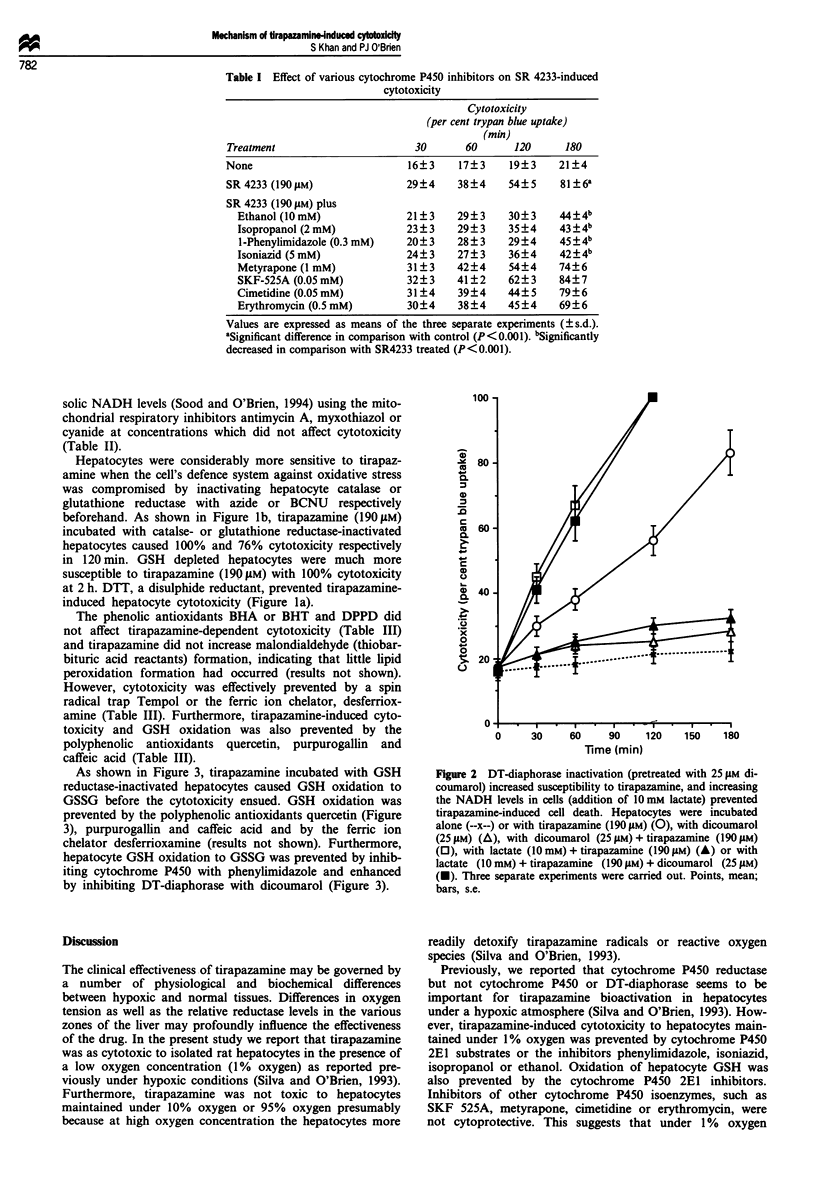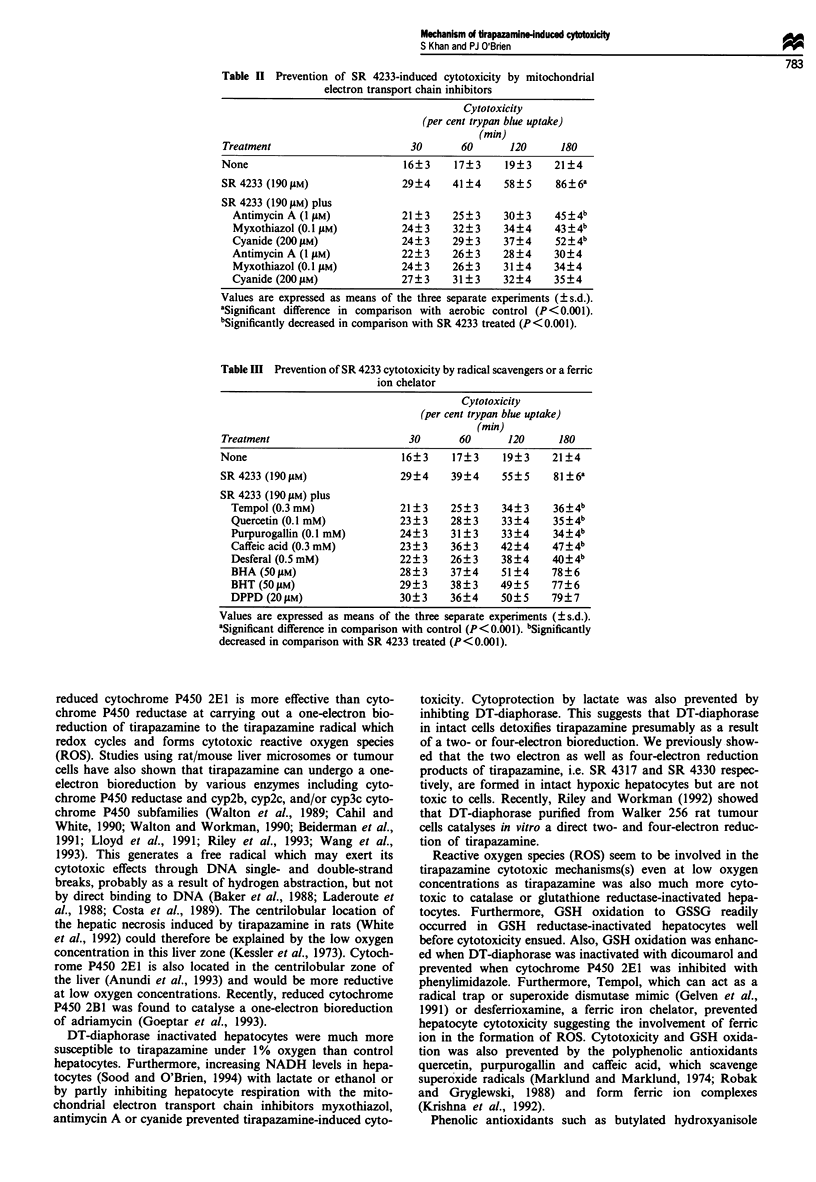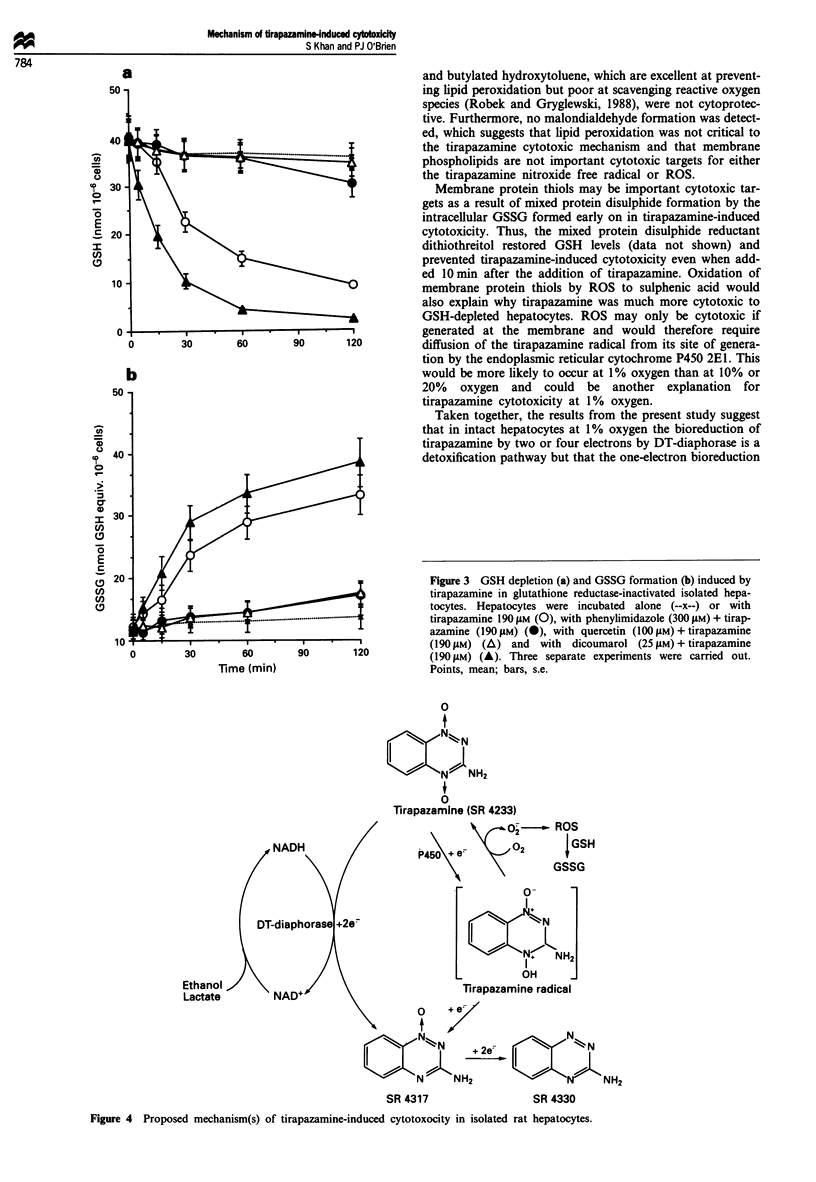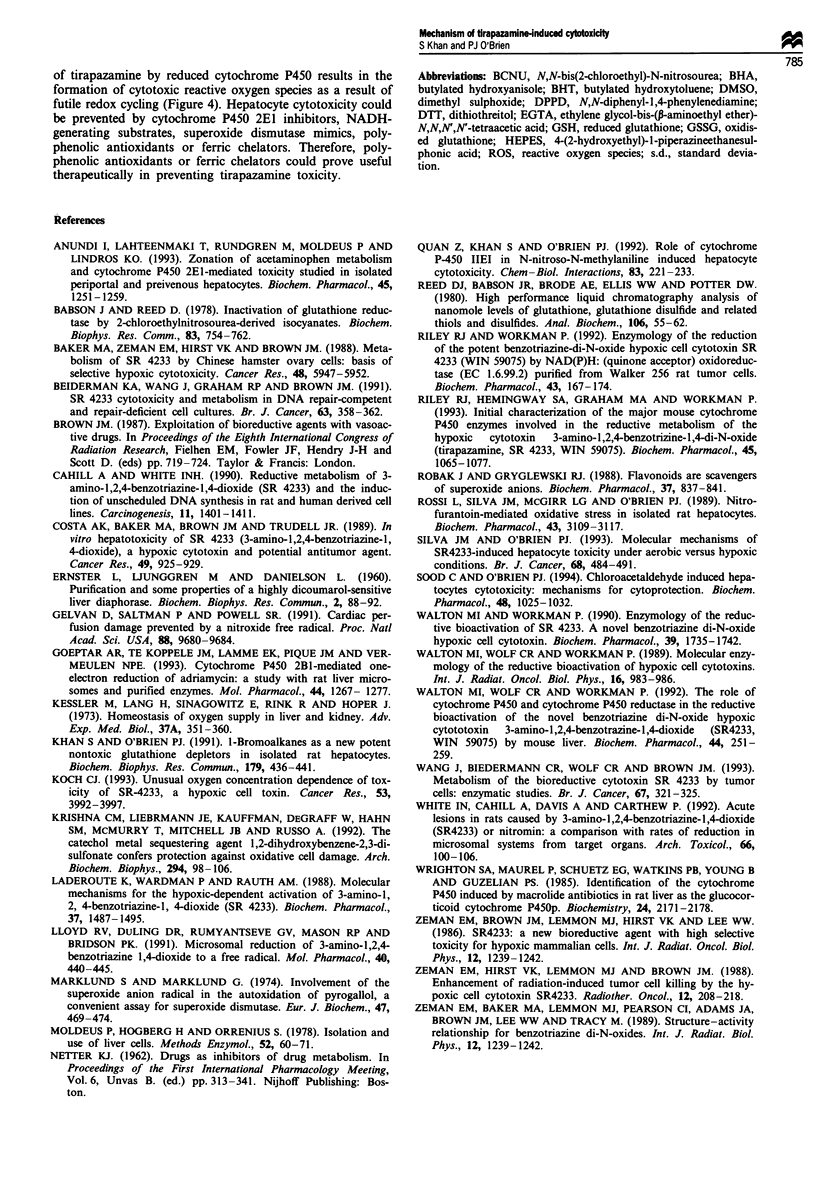# Molecular mechanisms of tirapazamine (SR 4233, Win 59075)-induced hepatocyte toxicity under low oxygen concentrations.

**DOI:** 10.1038/bjc.1995.151

**Published:** 1995-04

**Authors:** S. Khan, P. J. O'Brien

**Affiliations:** Faculty of Pharmacy, University of Toronto, Ontario, Canada.

## Abstract

Previously we showed that tirapazamine (SR 4233, Win 59075) is cytotoxic towards hepatocytes under conditions of hypoxia but not in 10% or 95% oxygen and that bioreduction by DT-diaphorase or cytochrome P450 is not a major pathway. In the present study, we report that tirapazamine is highly cytotoxic to isolated rat hepatocytes maintained under 1% oxygen and the molecular cytotoxic mechanism has been elucidated. Cytotoxicity was prevented by the cytochrome P450 2E1 inhibitors phenyl imidazole, isoniazid, isopropanol or ethanol, suggesting that cytochrome P450 2E1 catalysed tirapazamine reductive bioactivation. By contrast, dicoumarol, a DT-diaphorase inhibitor, markedly increased tirapazamine-induced cytotoxicity. Cytotoxicity was also inhibited in normal but not DT-diaphorase-inactivated hepatocytes by increasing cellular NADH levels with lactate or ethanol or the mitochondrial respiratory inhibitors. Evidence that oxygen activation contributed to cytotoxicity was that glutathione oxidation occurred well before cytotoxicity ensued and that tirapazamine was more cytotoxic towards catalase- or glutathione reductase-inactivated hepatocytes. Furthermore, polyphenolic antioxidants such as quercetin, caffeic acid or purpurogallin, the radical trap Tempol or the iron chelator desferrioxamine prevented tirapazamine-mediated cytotoxicity. However, the antioxidants diphenylphenylenediamine, butylated hydroxyanisole or butylated hydroxytoluene did not prevent cytotoxicity and malonaldehyde formation was not increased, suggesting that lipid peroxidation was not important. The above results suggest that DT-diaphorase detoxifies tirapazamine whereas reduced cytochrome P450 reduces tirapazamine to a nitrogen oxide anion radical which forms cytotoxic reactive oxygen species as a result of redox cycling.


British Journal of Cancer (1995) 71, 780–785
© 1995 Stockton Press Ltd All rights reserved 0007–0920/95 $12.00

### Molecular mechanisms of tirapazamine (SR 4233, WIN 59075)-induced hepatocyte toxicity under low oxygen concentrations

S Khan and PJ O'Brien

*Faculty of Pharmacy, University of Toronto, 19 Russell Street, Toronto, Ontario M5S 2S2, Canada.*

**Summary** Previously we showed that tirapazamine (SR 4233, Win 59075) is cytotoxic towards hepatocytes under conditions of hypoxia but not in 10% or 95% oxygen and that bioreduction by DT-diaphorase or cytochrome P450 is not a major pathway. In the present study, we report that tirapazamine is highly cytotoxic to isolated rat hepatocytes maintained under 1% oxygen and the molecular cytotoxic mechanism has been elucidated. Cytotoxicity was prevented by the cytochrome P450 2E1 inhibitors phenyl imidazole, isoniazid, isopropanol or ethanol, suggesting that cytochrome P450 2E1 catalysed tirapazamine reductive bioactivation. By contrast, dicoumarol, a DT-diaphorase inhibitor, markedly increased tirapazamine-induced cytotoxicity. Cytotoxicity was also inhibited in normal but not DT-diaphorase-inactivated hepatocytes by increasing cellular NADH levels with lactate or ethanol or the mitochondrial respiratory inhibitors. Evidence that oxygen activation contributed to cytotoxicity was that glutathione oxidation occurred well before cytotoxicity ensued and that tirapazamine was more cytotoxic towards catalase- or glutathione reductase-inactivated hepatocytes. Furthermore, polyphenolic antioxidants such as quercetin, caffeic acid or purpurogallin, the radical trap Tempol or the iron chelator desferrioxamine prevented tirapazamine-mediated cytotoxicity. However, the antioxidants diphenylphenylenediamine, butylated hydroxyanisole or butylated hydroxytoluene did not prevent cytotoxicity and malonaldehyde formation was not increased, suggesting that lipid peroxidation was not important. The above results suggest that DT-diaphorase detoxifies tirapazamine whereas reduced cytochrome P450 reduces tirapazamine to a nitrogen oxide anion radical which forms cytotoxic reactive oxygen species as a result of redox cycling.

**Keywords:** cytotoxicity; cytochrome P450 2E1; DT-diaphorase; tirapazamine; hepatocytes

Presently tirapazamine (3-amino-1, 2, 4-benzotriazine-1,4-di-*N*-oxide) is in clinical trials as a potential anti-tumour agent. It has been shown to be a highly selective hypoxic cell cytotoxin (Zeman *et al.*, 1986, 1989). It is also an effective anti-tumour agent *in vivo* in combination with radiation (Zeman *et al.*, 1988) or compounds which enhance tumour hypoxia (Brown, 1987). However subcapsular necrosis of the liver, necrosis of the kidney medulla and olfactory epithelium and bone marrow toxicity have been reported in rats after acute dosing with tirapazamine at 0.3 mmol $kg^{-1}$ (White *et al.*, 1992). Liver necrosis was confined to hepatocytes of the centrilobular zone (zone 3) which normally experience low oxygen tensions of 1–3% (White *et al.*, 1992). Recently, it has been confirmed that the toxicity of tirapazamine to Chinese hamster fibroblasts does not level off at high oxygen concentrations but continues to decrease as the oxygen concentrations increases (Koch, 1993). The hypoxic cytotoxicity ratio (HCR) for tirapazamine is 50–200 (Zeman *et al.*, 1986).

The molecular mechanism of tirapazamine cytotoxicity is believed to result from reductive bioactivation to cytotoxic radical intermediate (Baker *et al.*, 1988; Costa *et al.*, 1989; Laderoute *et al.*, 1988) and evidence for a nitroxide radical was obtained by electron spin resonance spectroscopy (Lloyd *et al.*, 1991). Enzymology studies with liver microsomes and NADPH have implicated cytochrome P450 and NADPH-cytochrome P450 reductase as the major hepatic reductases responsible for the reductive bioactivation of tirapazamine (Walton and Workman, 1990; Walton *et al.*, 1992; Riley *et al.*, 1993). Previously, using isolated rat hepatocytes as a non-proliferating model target cell and plasma membrane damage as the cytotoxic end point, we concluded that DT-diaphorase or reduced cytochrome P450 are not significantly involved in tirapazamine bioreduction in hypoxic hepatocytes (Silva and O'Brien, 1993). In the following we report that tirapazamine is just as cytotoxic to hepatocytes under 1% oxygen as was previously observed under nitrogen. Furthermore, in hepatocytes under 1% oxygen, tirapazamine was detoxified by DT-diaphorase and activated by cytochrome P450 2E1 as a result of a redox cycling-mediated oxygen activation. We also report for the first time that hepatocyte toxicity is prevented by the ferric chelator desferrioxamine, superoxide dismutase mimics and polyphenolic antioxidants that scavenge reactive oxygen species.

Correspondence: PJ O'Brien
Received 4 June 1994; revised 26 September 1994; accepted 16 November 1994

### Materials and methods

### *Chemicals*

Tirapazamine was a gift from Dr AM Rauth, Ontario Cancer Institute, Toronto, Ontario, Canada. Antimycin A, 1-bromoheptane, caffeic acid, cimetidine, dicoumarol dithiothreitol (DTT), erythromycin, reduced glutathione (GSH), oxidised glutathione (GSSG), isoniazid, lactic acid, metyrapone, myxothiazol, potassium cyanide, sodium azide and Tempol were obtained from Sigma (St Louis, MO, USA). 1-Phenylimidazole, purpurogallin, quercetin and trypan blue were obtained from Aldrich (Milwaukee, WI, USA). Desferrioxamine was a gift from Ciba Geigy Canada (Toronto, Ontario, Canada). *N,N*-bis(z-chloroethyl)-*N*-nitrosourea (BCNU) was a gift from Bristol-Myers (Syracuse, NY, USA). SKF-525A was a gift from Smith Kline Beecham (Oakville, Ontario, Canada). Collagenase (from *Clostridum histolyticum*) and HEPES were purchased from Boehringer-Mannheim (Montreal, PQ, Canada). High-performance liquid chromatography (HPLC)-grade solvents were purchased from Calden (Georgetown, Ontario, Canada). All other chemicals used were of analytical grade.

### *Animals*

Male Sprague–Dawley rats (body weight 250–300 g) fed a standard chow diet and tap water *ad libitum* were used to prepare hepatocytes.

*Isolation and incubation of hepatocytes*

The cells were isolated by collagenase perfusion of the liver as described by Moldeus *et al.* (1978). Routinely, 85–95% of the freshly isolated hepatocytes excluded trypan blue (trypan blue final concentration 0.2%). The cells ($1 \times 10^6$ cells $ml^{-1}$) were incubated in Krebs–Henseleit bicarbonate buffer (pH 7.4) supplemented with 12.5 mM HEPES under an atmosphere of 1% oxygen, 94% nitrogen and 5% carbon dioxide in 50 ml round-bottom flasks fitted on a standard-taper distillation adaptor for five flasks which was rotated (30 r.p.m.) on a rotary evaporator. The evaporator was positioned so that the axis of rotation deviated 45° from the water surface so that the flasks were immersed in the thermostated water. The gas mixture was supplied continuously to the surface of the incubation medium through the central vacuum exit of the evaporator. The oxygen level was monitored continuously with an oxygen electrode in the hepatocyte suspension and was $1.0 \pm 0.02\%$ ($10.5 \pm 1$ μM).

Tirapazamine was dissolved in dimethyl sulphoxide and added in a final concentration of 0.2% (v/v). The control incubation contained 0.2% (v/v) dimethyl sulphoxide alone. Glutathione-depleted hepatocytes were obtained by preincubating hepatocytes with 1-bromoheptane (200 μM) as previously described (Khan and O'Brien, 1991). To inactivate hepatocyte catalase (EC 1.11.1.6) and glutathione reductase (EC 1.6.4.2), sodium azide (final concentration 4 mM) and BCNU (final concentration, 50 μM) were added respectively to the cells 20 min before the start of the experiment (Babson and Reed, 1978; Rossi *et al.*, 1989). Azide and BCNU were not cytotoxic at these concentrations over a 4 h incubation period. To inactivate various cytochrome P450 isoenzymes, SKF-525A, ethanol, isopropanol, 1-phenylimidazole, isoniazid, metyrapone, cimetidine or erythromycin was added to the cells 10 min before the start of the experiment (Netter, 1962; Wrighton *et al.*, 1985; Quan *et al.*, 1992; Riley *et al.*, 1993). To inactivate DT-diaphorase, hepatocytes were preincubated with dicoumarol (final concentration 25 μM) for 10 min (Ernster *et al.*, 1960; Rossi *et al.*, 1989). Various antioxidants, radical scavengers, desferrioxamine (ferric ion chelator), artificial electron acceptors, DTT or mitochondrial electron transport chain inhibitors were preincubated for 5 min before the start of the experiment. All enzyme modifiers or other inhibitors were maintained in the cell medium throughout the experiment and were not cytotoxic at the concentrations used.

*Assays*

Hepatocyte viability was assessed by the trypan blue dye exclusion test in a Neubaur chamber by light microscopy. Viability was examined immediately after isolation, and at various time points during the experiment.

Total GSH and GSSG in the hepatocytes were measured by HPLC analysis in deproteinated samples (5% metaphosphoric acid) after derivatisation with iodoacetic acid and 1-fluoro-2,4-dinitrobenzene using a μ-Bondapak $NH_2$ column (Waters, Mississauga, Ontario, Canada) as described by Reed *et al.* (1980). GSH and GSSG were used as external standards. A Waters model 6000A solvent-delivery system equipped with a Waters model 660 solvent programmer, a WISP 710A automatic injector and a data module was used for analysis.

*Statistics*

Statistically significant differences between control and experimental groups were obtained using Student's *t*-test. The minimal level of significance chosen was $P<0.05$.

### Results

As shown in Figure 1, tirapazamine (190 μM) added to hepatocytes maintained under 1% oxygen/94% nitrogen/5% carbon dioxide caused 50% cytotoxicity within a 2 h incubation period. Untreated hepatocytes retained their viability under these conditions. The effect of various cytochrome P450 inhibitors on tirapazamine-induced cytotoxicity is presented in Table I. Cytochrome P450 2E1 inhibitors 1-phenylimidazole, isoniazid, isopropanol and ethanol were cytoprotective, whereas other cytochrome P450 inhibitors, metyrapone, SKF-525A, cimetidine and erythromycin, did not prevent tirapazamine-induced cytotoxicity. As shown in Figure 2, dicoumarol, a highly effective inhibitor of DT-diaphorase, increased the tirapazamine-dependent cytotoxicity at least 2-fold. None of the cytochrome P450 inhibitors or dicoumarol affected the viability of hepatocytes in the absence of tirapazamine. The rate of tirapazamine disappearance (2.1 nmol $min^{-1}$ $10^{-6}$ cells) in hepatocytes under 1% oxygen was a little slower than that found under a hypoxic environment (Silva and O'Brien, 1993).

Tirapazamine cytotoxicity was also prevented by increasing cytosolic NADH levels (Sood and O'Brien, 1994) by the addition of lactate or ethanol (Figure 2). This cytoprotection was prevented if hepatocyte DT-diaphorase was inhibited by dicoumarol (Figure 2). Cytotoxicity caused by tirapazamine was also markedly decreased by increasing hepatocyte cyto-

**Figure 1** (**a**) Increased susceptibility of GSH-depleted cells to tirapazamine. Hepatocytes were incubated alone (--x--), with 190 μM tirapazamine (○) or with DTT (5 mM) at 10 min + tirapazamine (190 μM) (▲). (△) GSH-depleted cells; (●) with tirapazamine (190 μM). Three separate experiments were carried out. Points, mean; bars, s.e. (**b**) Increased susceptibility of GSH reductase- (BCNU pretreated) or catalase (sodium azide pretreated)-inactivated cells to tirapazamine. Hepatocytes were incubated alone (--x--), with tirapazamine (190 μM) (○), with azide (4 mM) + tirapazamine (190 μM) (△), with BCNU (50 μM) + tirapazamine (190 μM) (●) with azide (4 mM) (■) or with BCNU (50 μM) (□). Three separate experiments were carried out. Points, mean; bars, s.e.

Table I Effect of various cytochrome P450 inhibitors on SR 4233-induced cytotoxicity

| Treatment | Cytotoxicity (per cent trypan blue uptake) (min) 30 | 60 | 120 | 180 |
|---|---|---|---|---|
| None | 16±3 | 17±3 | 19±3 | 21±4 |
| SR 4233 (190 μM) | 29±4 | 38±4 | 54±5 | 81±6[a] |
| SR 4233 (190 μM) plus | | | | |
| Ethanol (10 mM) | 21±3 | 29±3 | 30±3 | 44±4[b] |
| Isopropanol (2 mM) | 23±3 | 29±3 | 35±4 | 43±4[b] |
| 1-Phenylimidazole (0.3 mM) | 20±3 | 28±3 | 29±4 | 45±4[b] |
| Isoniazid (5 mM) | 24±3 | 27±3 | 36±4 | 42±4[b] |
| Metyrapone (1 mM) | 31±3 | 42±4 | 54±4 | 74±6 |
| SKF-525A (0.05 mM) | 32±3 | 41±2 | 62±3 | 84±7 |
| Cimetidine (0.05 mM) | 31±4 | 39±4 | 44±5 | 79±6 |
| Erythromycin (0.5 mM) | 30±4 | 38±4 | 45±4 | 69±6 |

Values are expressed as means of the three separate experiments (±s.d.). [a]Significant difference in comparison with control ($P<0.001$). [b]Significantly decreased in comparison with SR4233 treated ($P<0.001$).

solic NADH levels (Sood and O'Brien, 1994) using the mitochondrial respiratory inhibitors antimycin A, myxothiazol or cyanide at concentrations which did not affect cytotoxicity (Table II).

Hepatocytes were considerably more sensitive to tirapazamine when the cell's defence system against oxidative stress was compromised by inactivating hepatocyte catalase or glutathione reductase with azide or BCNU respectively beforehand. As shown in Figure 1b, tirapazamine (190 μM) incubated with catalse- or glutathione reductase-inactivated hepatocytes caused 100% and 76% cytotoxicity respectively in 120 min. GSH depleted hepatocytes were much more susceptible to tirapazamine (190 μM) with 100% cytotoxicity at 2 h. DTT, a disulphide reductant, prevented tirapazamine-induced hepatocyte cytotoxicity (Figure 1a).

The phenolic antioxidants BHA or BHT and DPPD did not affect tirapazamine-dependent cytotoxicity (Table III) and tirapazamine did not increase malondialdehyde (thiobarbituric acid reactants) formation, indicating that little lipid peroxidation formation had occurred (results not shown). However, cytotoxicity was effectively prevented by a spin radical trap Tempol or the ferric ion chelator, desferrioxamine (Table III). Furthermore, tirapazamine-induced cytotoxicity and GSH oxidation was also prevented by the polyphenolic antioxidants quercetin, purpurogallin and caffeic acid (Table III).

As shown in Figure 3, tirapazamine incubated with GSH reductase-inactivated hepatocytes caused GSH oxidation to GSSG before the cytotoxicity ensued. GSH oxidation was prevented by the polyphenolic antioxidants quercetin (Figure 3), purpurogallin and caffeic acid and by the ferric ion chelator desferrioxamine (results not shown). Furthermore, hepatocyte GSH oxidation to GSSG was prevented by inhibiting cytochrome P450 with phenylimidazole and enhanced by inhibiting DT-diaphorase with dicoumarol (Figure 3).

Figure 2 DT-diaphorase inactivation (pretreated with 25 μM dicoumarol) increased susceptibility to tirapazamine, and increasing the NADH levels in cells (addition of 10 mM lactate) prevented tirapazamine-induced cell death. Hepatocytes were incubated alone (--x--) or with tirapazamine (190 μM) (O), with dicoumarol (25 μM) (Δ), with dicoumarol (25 μM) + tirapazamine (190 μM) (□), with lactate (10 mM) + tirapazamine (190 μM) (▲) or with lactate (10 mM) + tirapazamine (190 μM) + dicoumarol (25 μM) (■). Three separate experiments were carried out. Points, mean; bars, s.e.

### Discussion

The clinical effectiveness of tirapazamine may be governed by a number of physiological and biochemical differences between hypoxic and normal tissues. Differences in oxygen tension as well as the relative reductase levels in the various zones of the liver may profoundly influence the effectiveness of the drug. In the present study we report that tirapazamine was as cytotoxic to isolated rat hepatocytes in the presence of a low oxygen concentration (1% oxygen) as reported previously under hypoxic conditions (Silva and O'Brien, 1993). Furthermore, tirapazamine was not toxic to hepatocytes maintained under 10% oxygen or 95% oxygen presumably because at high oxygen concentration the hepatocytes more readily detoxify tirapazamine radicals or reactive oxygen species (Silva and O'Brien, 1993).

Previously, we reported that cytochrome P450 reductase but not cytochrome P450 or DT-diaphorase seems to be important for tirapazamine bioactivation in hepatocytes under a hypoxic atmosphere (Silva and O'Brien, 1993). However, tirapazamine-induced cytotoxicity to hepatocytes maintained under 1% oxygen was prevented by cytochrome P450 2E1 substrates or the inhibitors phenylimidazole, isoniazid, isopropanol or ethanol. Oxidation of hepatocyte GSH was also prevented by the cytochrome P450 2E1 inhibitors. Inhibitors of other cytochrome P450 isoenzymes, such as SKF 525A, metyrapone, cimetidine or erythromycin, were not cytoprotective. This suggests that under 1% oxygen

**Table II** Prevention of SR 4233-induced cytotoxicity by mitochondrial electron transport chain inhibitors

| | *Cytotoxicity (per cent trypan blue uptake) (min)* | | | |
|---|---|---|---|---|
| *Treatment* | *30* | *60* | *120* | *180* |
| None | 16±3 | 17±3 | 19±3 | 21±4 |
| SR 4233 (190 μM) | 29±4 | 41±4 | 58±5 | 86±6[a] |
| SR 4233 (190 μM) plus | | | | |
| Antimycin A (1 μM) | 21±3 | 25±3 | 30±3 | 45±4[b] |
| Myxothiazol (0.1 μM) | 24±3 | 32±3 | 34±4 | 43±4[b] |
| Cyanide (200 μM) | 24±3 | 29±3 | 37±4 | 52±4[b] |
| Antimycin A (1 μM) | 22±3 | 26±3 | 28±4 | 30±4 |
| Myxothiazol (0.1 μM) | 24±3 | 26±3 | 31±4 | 34±4 |
| Cyanide (200 μM) | 27±3 | 31±3 | 32±4 | 35±4 |

Values are expressed as means of the three separate experiments (±s.d.). [a]Significant difference in comparison with aerobic control ($P<0.001$). [b]Significantly decreased in comparison with SR 4233 treated ($P<0.001$).

**Table III** Prevention of SR 4233 cytotoxicity by radical scavengers or a ferric ion chelator

| | *Cytotoxicity (per cent trypan blue uptake) (min)* | | | |
|---|---|---|---|---|
| *Treatment* | *30* | *60* | *120* | *180* |
| None | 16±3 | 17±3 | 19±3 | 21±4 |
| SR 4233 (190 μM) | 29±4 | 39±4 | 55±5 | 81±6[a] |
| SR 4233 (190 μM) plus | | | | |
| Tempol (0.3 mM) | 21±3 | 25±3 | 34±3 | 36±4[b] |
| Quercetin (0.1 mM) | 23±3 | 28±3 | 33±4 | 35±4[b] |
| Purpurogallin (0.1 mM) | 24±3 | 31±3 | 33±4 | 34±4[b] |
| Caffeic acid (0.3 mM) | 23±3 | 36±3 | 42±4 | 47±4[b] |
| Desferal (0.5 mM) | 22±3 | 26±3 | 38±4 | 40±4[b] |
| BHA (50 μM) | 28±3 | 37±4 | 51±4 | 78±6 |
| BHT (50 μM) | 29±3 | 38±3 | 49±5 | 77±6 |
| DPPD (20 μM) | 30±3 | 36±4 | 50±5 | 79±7 |

Values are expressed as means of the three separate experiments (±s.d.). [a]Significant difference in comparison with control ($P<0.001$). [b]Significantly decreased in comparison with SR 4233 treated ($P<0.001$).

reduced cytochrome P450 2E1 is more effective than cytochrome P450 reductase at carrying out a one-electron bioreduction of tirapazamine to the tirapazamine radical which redox cycles and forms cytotoxic reactive oxygen species (ROS). Studies using rat/mouse liver microsomes or tumour cells have also shown that tirapazamine can undergo a one-electron bioreduction by various enzymes including cytochrome P450 reductase and cyp2b, cyp2c, and/or cyp3c cytochrome P450 subfamilies (Walton *et al.*, 1989; Cahil and White, 1990; Walton and Workman, 1990; Beiderman *et al.*, 1991; Lloyd *et al.*, 1991; Riley *et al.*, 1993; Wang *et al.*, 1993). This generates a free radical which may exert its cytotoxic effects through DNA single- and double-strand breaks, probably as a result of hydrogen abstraction, but not by direct binding to DNA (Baker *et al.*, 1988; Laderoute *et al.*, 1988; Costa *et al.*, 1989). The centrilobular location of the hepatic necrosis induced by tirapazamine in rats (White *et al.*, 1992) could therefore be explained by the low oxygen concentration in this liver zone (Kessler *et al.*, 1973). Cytochrome P450 2E1 is also located in the centrilobular zone of the liver (Anundi *et al.*, 1993) and would be more reductive at low oxygen concentrations. Recently, reduced cytochrome P450 2B1 was found to catalyse a one-electron bioreduction of adriamycin (Goeptar *et al.*, 1993).

DT-diaphorase inactivated hepatocytes were much more susceptible to tirapazamine under 1% oxygen than control hepatocytes. Furthermore, increasing NADH levels in hepatocytes (Sood and O'Brien, 1994) with lactate or ethanol or by partly inhibiting hepatocyte respiration with the mitochondrial electron transport chain inhibitors myxothiazol, antimycin A or cyanide prevented tirapazamine-induced cytotoxicity. Cytoprotection by lactate was also prevented by inhibting DT-diaphorase. This suggests that DT-diaphorase in intact cells detoxifies tirapazamine presumably as a result of a two- or four-electron bioreduction. We previously showed that the two electron as well as four-electron reduction products of tirapazamine, i.e. SR 4317 and SR 4330 respectively, are formed in intact hypoxic hepatocytes but are not toxic to cells. Recently, Riley and Workman (1992) showed that DT-diaphorase purified from Walker 256 rat tumour cells catalyses *in vitro* a direct two- and four-electron reduction of tirapazamine.

Reactive oxygen species (ROS) seem to be involved in the tirapazamine cytotoxic mechanisms(s) even at low oxygen concentrations as tirapazamine was also much more cytotoxic to catalase or glutathione reductase-inactivated hepatocytes. Furthermore, GSH oxidation to GSSG readily occurred in GSH reductase-inactivated hepatocytes well before cytotoxicity ensued. Also, GSH oxidation was enhanced when DT-diaphorase was inactivated with dicoumarol and prevented when cytochrome P450 2E1 was inhibited with phenylimidazole. Furthermore, Tempol, which can act as a radical trap or superoxide dismutase mimic (Gelven *et al.*, 1991) or desferrioxamine, a ferric iron chelator, prevented hepatocyte cytotoxicity suggesting the involvement of ferric ion in the formation of ROS. Cytotoxicity and GSH oxidation was also prevented by the polyphenolic antioxidants quercetin, purpurogallin and caffeic acid, which scavenge superoxide radicals (Marklund and Marklund, 1974; Robak and Gryglewski, 1988) and form ferric ion complexes (Krishna *et al.*, 1992).

Phenolic antioxidants such as butylated hydroxyanisole

Figure 3 GSH depletion (a) and GSSG formation (b) induced by tirapazamine in glutathione reductase-inactivated isolated hepatocytes. Hepatocytes were incubated alone (--x--) or with tirapazamine 190 μM (O), with phenylimidazole (300 μM) + tirapazamine (190 μM) (●), with quercetin (100 μM) + tirapazamine (190 μM) (Δ) and with dicoumarol (25 μM) + tirapazamine (190 μM) (▲). Three separate experiments were carried out. Points, mean; bars, s.e.

and butylated hydroxytoluene, which are excellent at preventing lipid peroxidation but poor at scavenging reactive oxygen species (Robek and Gryglewski, 1988), were not cytoprotective. Furthermore, no malondialdehyde formation was detected, which suggests that lipid peroxidation was not critical to the tirapazamine cytotoxic mechanism and that membrane phospholipids are not important cytotoxic targets for either the tirapazamine nitroxide free radical or ROS.

Membrane protein thiols may be important cytotoxic targets as a result of mixed protein disulphide formation by the intracellular GSSG formed early on in tirapazamine-induced cytotoxicity. Thus, the mixed protein disulphide reductant dithiothreitol restored GSH levels (data not shown) and prevented tirapazamine-induced cytotoxicity even when added 10 min after the addition of tirapazamine. Oxidation of membrane protein thiols by ROS to sulphenic acid would also explain why tirapazamine was much more cytotoxic to GSH-depleted hepatocytes. ROS may only be cytotoxic if generated at the membrane and would therefore require diffusion of the tirapazamine radical from its site of generation by the endoplasmic reticular cytochrome P450 2E1. This would be more likely to occur at 1% oxygen than at 10% or 20% oxygen and could be another explanation for tirapazamine cytotoxicity at 1% oxygen.

Taken together, the results from the present study suggest that in intact hepatocytes at 1% oxygen the bioreduction of tirapazamine by two or four electrons by DT-diaphorase is a detoxification pathway but that the one-electron bioreduction

Figure 4 Proposed mechanism(s) of tirapazamine-induced cytotoxocity in isolated rat hepatocytes.

of tirapazamine by reduced cytochrome P450 results in the formation of cytotoxic reactive oxygen species as a result of futile redox cycling (Figure 4). Hepatocyte cytotoxicity could be prevented by cytochrome P450 2E1 inhibitors, NADH-generating substrates, superoxide dismutase mimics, polyphenolic antioxidants or ferric chelators. Therefore, polyphenolic antioxidants or ferric chelators could prove useful therapeutically in preventing tirapazamine toxicity.

**Abbreviations:** BCNU, *N,N*-bis(2-chloroethyl)-N-nitrosourea; BHA, butylated hydroxyanisole; BHT, butylated hydroxytoluene; DMSO, dimethyl sulphoxide; DPPD, *N,N*-diphenyl-1,4-phenylenediamine; DTT, dithiothreitol; EGTA, ethylene glycol-bis-(β-aminoethyl ether)-*N,N,N',N'*-tetraacetic acid; GSH, reduced glutathione; GSSG, oxidised glutathione; HEPES, 4-(2-hydroxyethyl)-1-piperazineethanesulphonic acid; ROS, reactive oxygen species; s.d., standard deviation.

### References

ANUNDI I, LAHTEENMAKI T, RUNDGREN M, MOLDEUS P AND LINDROS KO. (1993). Zonation of acetaminophen metabolism and cytochrome P450 2E1-mediated toxicity studied in isolated periportal and preivenous hepatocytes. *Biochem. Pharmacol.*, **45**, 1251-1259.

BABSON J AND REED D. (1978). Inactivation of glutathione reductase by 2-chloroethylnitrosourea-derived isocyanates. *Biochem. Biophys. Res. Comm.*, **83**, 754-762.

BAKER MA, ZEMAN EM, HIRST VK AND BROWN JM. (1988). Metabolism of SR 4233 by Chinese hamster ovary cells: basis of selective hypoxic cytotoxicity. *Cancer Res.*, **48**, 5947-5952.

BEIDERMAN KA, WANG J, GRAHAM RP AND BROWN JM. (1991). SR 4233 cytotoxicity and metabolism in DNA repair-competent and repair-deficient cell cultures. *Br. J. Cancer*, **63**, 358-362.

BROWN JM. (1987). Exploitation of bioreductive agents with vasoactive drugs. In *Proceedings of the Eighth International Congress of Radiation Research*, Fielhen EM, Fowler JF, Hendry J-H and Scott D. (eds) pp. 719-724. Taylor & Francis: London.

CAHILL A AND WHITE INH. (1990). Reductive metabolism of 3-amino-1,2,4-benzotriazine-1,4-dioxide (SR 4233) and the induction of unscheduled DNA synthesis in rat and human derived cell lines. *Carcinogenesis*, **11**, 1401-1411.

COSTA AK, BAKER MA, BROWN JM AND TRUDELL JR. (1989). *In vitro* hepatotoxicity of SR 4233 (3-amino-1,2,4-benzotriazine-1, 4-dioxide), a hypoxic cytotoxin and potential antitumor agent. *Cancer Res.*, **49**, 925-929.

ERNSTER L, LJUNGGREN M AND DANIELSON L. (1960). Purification and some properties of a highly dicoumarol-sensitive liver diaphorase. *Biochem. Biophys. Res. Commun.*, **2**, 88-92.

GELVAN D, SALTMAN P AND POWELL SR. (1991). Cardiac perfusion damage prevented by a nitroxide free radical. *Proc. Natl Acad. Sci. USA*, **88**, 9680-9684.

GOEPTAR AR, TE KOPPELE JM, LAMME EK, PIQUE JM AND VERMEULEN NPE. (1993). Cytochrome P450 2B1-mediated one-electron reduction of adriamycin: a study with rat liver microsomes and purified enzymes. *Mol. Pharmacol.*, **44**, 1267- 1277.

KESSLER M, LANG H, SINAGOWITZ E, RINK R AND HOPER J. (1973). Homeostasis of oxygen supply in liver and kidney. *Adv. Exp. Med. Biol.*, **37A**, 351-360.

KHAN S AND O'BRIEN PJ. (1991). 1-Bromoalkanes as a new potent nontoxic glutathione depletors in isolated rat hepatocytes. *Biochem. Biophys. Res. Commun.*, **179**, 436-441.

KOCH CJ. (1993). Unusual oxygen concentration dependence of toxicity of SR-4233, a hypoxic cell toxin. *Cancer Res.*, **53**, 3992-3997.

KRISHNA CM, LIEBRMANN JE, KAUFFMAN, DEGRAFF W, HAHN SM, MCMURRY T, MITCHELL JB AND RUSSO A. (1992). The catechol metal sequestering agent 1,2-dihydroxybenzene-2,3-disulfonate confers protection against oxidative cell damage. *Arch. Biochem. Biophys.*, **294**, 98-106.

LADEROUTE K, WARDMAN P AND RAUTH AM. (1988). Molecular mechanisms for the hypoxic-dependent activation of 3-amino-1, 2, 4-benzotriazine-1, 4-dioxide (SR 4233). *Biochem. Pharmacol.*, **37**, 1487-1495.

LLOYD RV, DULING DR, RUMYANTSEVE GV, MASON RP AND BRIDSON PK. (1991). Microsomal reduction of 3-amino-1,2,4-benzotriazine 1,4-dioxide to a free radical. *Mol. Pharmacol.*, **40**, 440-445.

MARKLUND S AND MARKLUND G. (1974). Involvement of the superoxide anion radical in the autoxidation of pyrogallol, a convenient assay for superoxide dismutase. *Eur. J. Biochem.*, **47**, 469-474.

MOLDEUS P, HOGBERG H AND ORRENIUS S. (1978). Isolation and use of liver cells. *Methods Enzymol.*, **52**, 60-71.

NETTER KJ. (1962). Drugs as inhibitors of drug metabolism. In *Proceedings of the First International Pharmacology Meeting*, Vol. 6, Unvas B. (ed.) pp. 313-341. Nijhoff Publishing: Boston.

QUAN Z, KHAN S AND O'BRIEN PJ. (1992). Role of cytochrome P-450 IIEI in N-nitroso-N-methylaniline induced hepatocyte cytotoxicity. *Chem-Biol. Interactions*, **83**, 221-233.

REED DJ, BABSON JR, BRODE AE, ELLIS WW AND POTTER DW. (1980). High performance liquid chromatography analysis of nanomole levels of glutathione, glutathione disulfide and related thiols and disulfides. *Anal. Biochem.*, **106**, 55-62.

RILEY RJ AND WORKMAN P. (1992). Enzymology of the reduction of the potent benzotriazine-di-N-oxide hypoxic cell cytotoxin SR 4233 (WIN 59075) by NAD(P)H: (quinone acceptor) oxidoreductase (EC 1.6.99.2) purified from Walker 256 rat tumor cells. *Biochem. Pharmacol.*, **43**, 167-174.

RILEY RJ, HEMINGWAY SA, GRAHAM MA AND WORKMAN P. (1993). Initial characterization of the major mouse cytochrome P450 enzymes involved in the reductive metabolism of the hypoxic cytotoxin 3-amino-1,2,4-benzotrizine-1,4-di-N-oxide (tirapazamine, SR 4233, WIN 59075). *Biochem. Pharmacol.*, **45**, 1065-1077.

ROBAK J AND GRYGLEWSKI RJ. (1988). Flavonoids are scavengers of superoxide anions. *Biochem. Pharmacol.*, **37**, 837-841.

ROSSI L, SILVA JM, MCGIRR LG AND O'BRIEN PJ. (1989). Nitrofurantoin-mediated oxidative stress in isolated rat hepatocytes. *Biochem. Pharmacol.*, **43**, 3109-3117.

SILVA JM AND O'BRIEN PJ. (1993). Molecular mechanisms of SR4233-induced hepatocyte toxicity under aerobic versus hypoxic conditions. *Br. J. Cancer*, **68**, 484-491.

SOOD C AND O'BRIEN PJ. (1994). Chloroacetaldehyde induced hepatocytes cytotoxicity: mechanisms for cytoprotection. *Biochem. Pharmacol.*, **48**, 1025-1032.

WALTON MI AND WORKMAN P. (1990). Enzymology of the reductive bioactivation of SR 4233. A novel benzotriazine di-N-oxide hypoxic cell cytotoxin. *Biochem. Pharmacol.*, **39**, 1735-1742.

WALTON MI, WOLF CR AND WORKMAN P. (1989). Molecular enzymology of the reductive bioactivation of hypoxic cell cytotoxins. *Int. J. Radiat. Oncol. Biol. Phys.*, **16**, 983-986.

WALTON MI, WOLF CR AND WORKMAN P. (1992). The role of cytochrome P450 and cytochrome P450 reductase in the reductive bioactivation of the novel benzotriazine di-N-oxide hypoxic cytototoxin 3-amino-1,2,4-benzotrazine-1,4-dioxide (SR4233, WIN 59075) by mouse liver. *Biochem. Pharmacol.*, **44**, 251-259.

WANG J, BIEDERMANN CR, WOLF CR AND BROWN JM. (1993). Metabolism of the bioreductive cytotoxin SR 4233 by tumor cells: enzymatic studies. *Br. J. Cancer*, **67**, 321-325.

WHITE IN, CAHILL A, DAVIS A AND CARTHEW P. (1992). Acute lesions in rats caused by 3-amino-1,2,4-benzotriazine-1,4-dioxide (SR4233) or nitromin: a comparison with rates of reduction in microsomal systems from target organs. *Arch. Toxicol.*, **66**, 100-106.

WRIGHTON SA, MAUREL P, SCHUETZ EG, WATKINS PB, YOUNG B AND GUZELIAN PS. (1985). Identification of the cytochrome P450 induced by macrolide antibiotics in rat liver as the glucocorticoid cytochrome P450p. *Biochemistry*, **24**, 2171-2178.

ZEMAN EM, BROWN JM, LEMMON MJ, HIRST VK AND LEE WW. (1986). SR4233: a new bioreductive agent with high selective toxicity for hypoxic mammalian cells. *Int. J. Radiat. Oncol. Biol. Phys.*, **12**, 1239-1242.

ZEMAN EM, HIRST VK, LEMMON MJ AND BROWN JM. (1988). Enhancement of radiation-induced tumor cell killing by the hypoxic cell cytotoxin SR4233. *Radiother. Oncol.*, **12**, 208-218.

ZEMAN EM, BAKER MA, LEMMON MJ, PEARSON CI, ADAMS JA, BROWN JM, LEE WW AND TRACY M. (1989). Structure-activity relationship for benzotriazine di-N-oxides. *Int. J. Radiat. Biol. Phys.*, **12**, 1239-1242.